# Complete Genome Sequence of *Listeria monocytogenes* N2306, a Strain Associated with the 2013-2014 Listeriosis Outbreak in Switzerland

**DOI:** 10.1128/genomeA.00553-15

**Published:** 2015-05-28

**Authors:** Taurai Tasara, Rebecca Ebner, Jochen Klumpp, Roger Stephan

**Affiliations:** a Institute for Food Safety and Hygiene, Vetsuisse Faculty, University of Zurich, Zurich, Switzerland; b Institute of Food, Nutrition and Health, ETH Zurich, Zurich, Switzerland

## Abstract

We present the complete genome sequence of *Listeria monocytogenes* N2306, a serotype 4b clinical strain isolated during the 2013-2014 nationwide listeriosis outbreak in Switzerland.

## GENOME ANNOUNCEMENT

The Gram-positive foodborne pathogen *Listeria monocytogenes* causes listeriosis, which is a rare but serious foodborne disease associated with severe life-threatening illnesses and high mortality among those with weakened immune systems (1). Switzerland has previously experienced serious large-scale listeriosis outbreaks, and more recently a nationwide outbreak occurred that was linked to the consumption of contaminated ready-to-eat salads (2–5). We have determined the complete genome sequence of *L. monocytogenes* N2306, a serotype 4b strain responsible for this outbreak.

Genomic DNA isolated from N2306 was subjected to single-molecule real-time sequencing on a Pacific Biosciences RS2 device (10-kb insert library, P4/C2 chemistry) at the Functional Genomics Centre Zurich (FGCZ). A total of 131,245 sequence reads (126-fold genome coverage) with an average length of 3,261 kb were generated. The N2306 genome was assembled *de novo* into a 2,911,639-bp single chromosome with a 38% GC content using the SMRT Analysis version 2.1.2 software and the HGAP3 algorithm. Gene prediction and annotation of the genome were carried out using the NCBI Prokaryotic Genomes Automatic Annotation Pipeline (http://www.ncbi.nlm.nih.gov/genome/annotation_prok).

The N2306 genome comprises 2,885 open reading frames, including 2,781 coding sequences (CDS), 18 pseudogenes, 67 tRNA genes, and 6 rRNA operons. An incomplete prophage at position 235,349 to 258,324 (22,975 bp) of the genome was detected using the Phage search tool (PHAST [6]). N2306 was assigned to sequence type 4 (ST4) and grouped into clonal complex 4 (CC4) using multilocus sequence typing (MLST) analysis (7) (http://www.pasteur.fr/recherche/genopole/PF8/mlst/index.html).

The availability of the N2306 genome will enable its comparison with other genomes of listeriosis outbreak strains, providing more insights into virulence factors and stress resistance genetic features associated with this important foodborne pathogen.

### Nucleotide sequence accession number. 

The complete N2306 genome sequence has been deposited in GenBank under the accession number CP011004.
